# Cortex Mori Radicis Extract promotes neurite outgrowth of diabetic rats by activating PI3K/AKT signaling and inhibiting Ca^2+^ influx associated with upregulation of transient receptor potential canonical channel 1

**DOI:** 10.3892/mmr.2020.11008

**Published:** 2020-02-28

**Authors:** Min Lu, Tao Yi, Yong Xiong, Qian Wang, Nina Yin

Mol Med Rep 21: 320-328, 2020; DOI: 10.3892/mmr.2019.10839

Subsequently to the publication of the above paper, the authors have realized that Fig. 6E and F contained errors. Essentially, some of the data shown in Fig. 6E were incorrect, and consequently, the quantification of the data illustrated in Fig. 6F were likewise incorrect. The corrected version of Fig. 6, showing the correct data for these figure parts, is shown opposite.

Note that these errors in the data selection for this figure did not seriously affect the overall conclusions reported in the study. The authors are grateful to the Editor for allowing them the opportunity to publish this Corrigendum, and wish to apologize to the readership of the Journal for any inconvenience caused.

## Figures and Tables

**Figure 6.**
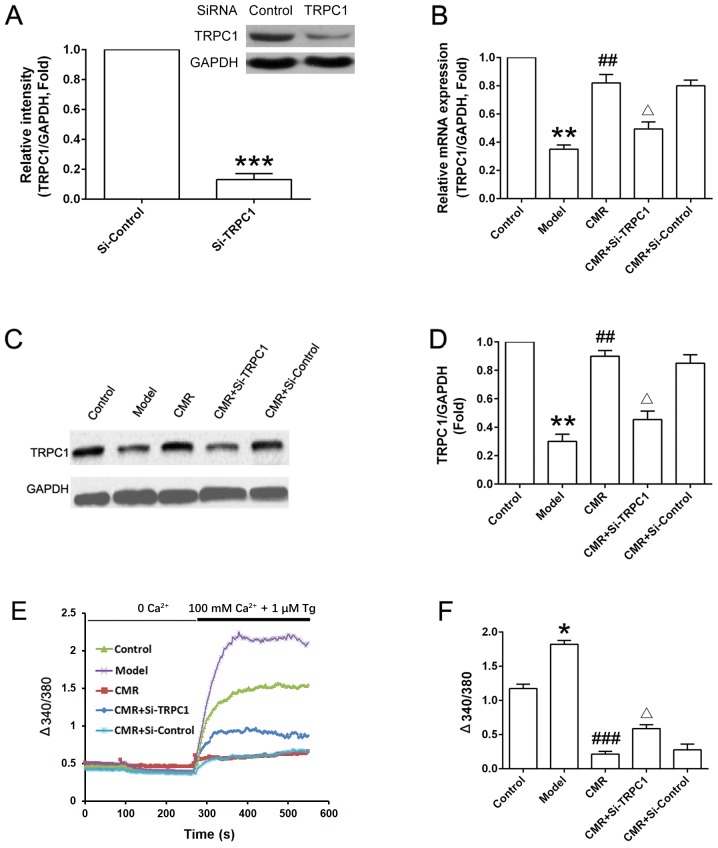
Effect of CMR on the TRPC1/Ca^2+^ axis in the DRG neurons of diabetic rats. DRG neurons were seeded in a 6- well plate (5×105 cells/well) and were cultured for 24 h. (A) Normal rats were treated with control siRNA and TRPC1 siRNA. DRG neurons were collected and TRPC1 expression was detected by western blotting and was statistically analyzed. (B) mRNA expression levels of TRPC1 in the DRG neurons from different groups were detected by reverse transcription- quantitative PCR and analyzed. (C) Protein expression levels of TRPC1 in the DRG neurons from different groups were detected by western blotting and (D) were statistically analyzed. GAPDH was used to confirm equal sample loading. (E) DRG neurons were seeded in a 6- well plate (5×105 cells/well) and were cultured for 24 h. Cytosolic Ca^2+^ was measured in Fura- 2- loaded DRG neurons and (F) statistically analyzed (340/380). Representative traces of intracellular Ca^2+^ in DRG neurons are shown. *P<0.05, **P<0.01, ***P<0.001 vs. control; ##P<0.01 vs. model; ΔP<0.05 vs. CMR. CMR, Cortex Mori Radicis extract; TRPC1, transient receptor potential canonical channel 1; si-, small interfering RNA; DRG, dorsal root ganglia; Tg, thapsigargin.

